# Weight, Weight Perceptions, and Health and Well-Being Among Canadian Adolescents: Evidence From the 2017-2018 Canadian Community Health Survey

**DOI:** 10.1177/08901171211031064

**Published:** 2021-07-20

**Authors:** Lei Chai, Jia Xue

**Affiliations:** 1 Department of Sociology, University of Toronto, Toronto, Ontario, Canada; 2 Factor-Inwentash Faculty of Social Work & Faculty of Information, University of Toronto, Toronto, Ontario, Canada

**Keywords:** obesity, weight management, health behavior

## Abstract

**Purpose:**

The present study examines the extent to which (mis)matched weight and weight perceptions predict adolescents’ self-rated health, mental health, and life satisfaction.

**Design:**

Quantitative, cross-sectional study.

**Setting:**

Data from the 2017-2018 Canadian Community Health Survey (CCHS)—a nationally representative sample collected by Statistics Canada.

**Participants:**

Canadian adolescents aged between 12 and 17 (n = 8,081).

**Measures:**

The dependent variables are self-rated health, mental health, and life satisfaction. The independent variable is (mis)matched weight and weight perceptions.

**Analysis:**

We perform a series of ordinary least squares (OLS) regression models.

**Results:**

Overweight adolescents with overweight perceptions are associated with poorer self-rated health (b = −.546, p < .001 for boys; b = −.476, p < .001 for girls), mental health (b = −.278, p < .001 for boys; b = −.433, p < .001 for girls), and life satisfaction (b = −.544, p < .001 for boys; b = −.617, p < .001 for girls) compared to their counterparts with normal weight and normal weight perceptions. Similar patterns have also been observed among normal weight adolescents with overweight perceptions (e.g., normal weight adolescents with overweight perceptions are associated with poorer self-rated health (b = −.541, p < .01 for boys; b = −.447, p < .001 for girls)).

**Conclusion:**

Normal weight adolescents are not immune to adverse self-rated health, mental health, and life satisfaction because their weight perceptions are also a contributing factor to health and well-being consequences.

## Purpose

The high rates of overweight and obesity continue to be an important public health concern among adolescents.^1^ In Canada, 24.5% of adolescents aged between 12 and 17 report being overweight or obese.^2^ Prior research has well-established that overweight and obesity are negatively associated with adolescents’ health and well-being.^3-6^ Some scholars have also recognized the importance of weight perceptions while assessing adolescents’ health and well-being disparities.^7-9^ Although these two separate bodies of research have contributed to the literature in significant ways, scholars have yet to explore whether (mis)matched weight and weight perceptions might be linked to greater health and well-being disparities among adolescents, with one exception documenting depressive symptoms in the United States.^10^

With this gap in mind, the present study selects Canadian adolescents aged between 12 and 17 from a nationally representative sample—the 2017-2018 Canadian Community Health Survey (CCHS)—to examine the extent to which (mis)matched weight and weight perceptions predict adolescents’ self-rated health, mental health, and life satisfaction. Using theories of double jeopardy and health congruency as guiding frameworks,^10^ we seek to test two competing explanations about whether and how the combinations of weight and weight perceptions might matter for adolescents’ health and well-being. To organize the conceptual and theoretical rationale behind our study, we create different scenarios of actual and perceived weight combinations (see Table 1).

**Table 1.** Scenarios of the Combinations Between Weight and Weight Perceptions.

	Underweight perceptions	Normal weight perceptions	Overweight perceptions
Underweight	Too few cases (excluded)	Weight congruency (lower levels of health and well-being)	Too few cases (excluded)
Normal weight	Weight congruency (lower levels of health and well-being)	Reference group (higher levels of health and well-being)	Weight congruency (lower levels of health and well-being)
Overweight	Too few cases (excluded)	Weight congruency (lower levels of health and well-being)	Double jeopardy (lower levels of health and well-being)

A large body of research has documented the effect of weight on adolescents’ health and well-being. According to the stress process model,^11^ the stigmatized physical and social statuses attached to overweight and obesity can be stressful, which might limit one’s ability to cope, thereby contributing to worse health and well-being outcomes. An extensive research has found evidence supporting this claim.^3-6^ For instance, one study based on a meta-analysis shows that overweight and obese adolescents are more likely to report worse physical health outcomes.^12^ Likewise, a recent review piece demonstrates that overweight and obese adolescents are associated with elevated mental health problems such as depression and anxiety.^13^ There is also evidence that overweight and obese adolescents are linked to lower levels of life satisfaction.^14^

In addition to actual weight, some scholars have also recognized the importance of weight perceptions while assessing health and well-being disparities among adolescents. Weight perceptions have been conceptualized as powerful self-schemas, which are developed based on social norms and cultural values.^15^ The burden of negative self-evaluation associated with overweight perceptions might lead to adverse consequences.^10^ Past research has found that adolescents with overweight perceptions are associated with worse health and well-being outcomes.^7-9^ For instance, a recent review piece suggests that overweight perceptions are associated with elevated levels of psychological distress. Similarly, overweight perceptions are also linked to worse physical health outcomes attributed to lack of health-promoting behaviors.^16^ Likewise, using data from a large online survey conducted in 2016, one study shows that overweight perceptions are associated with lower levels of life satisfaction.^17^

Despite these valuable findings, little is known about whether (mis)matched weight and weight perceptions might matter for adolescents’ health and well-being. Based on double jeopardy and health congruency theories, we seek to develop two competing explanations that help understand the potential relationship between (mis)matched weight and weight perceptions and adolescents’ health and well-being.^10^ Consistent with the prediction of the stress process model,^11^ double jeopardy theory posits that adolescents with multiple disadvantaged social statuses face an accumulation of stressors, which might lead to greater exposure to health and well-being problems compared to their counterparts with fewer disadvantaged statuses.^18,19^ Given that overweight and overweight perceptions each represent a stigmatized status, overweight adolescents with overweight perceptions might be at greater risk of health problems.^10^ By contrast, health congruency theory posits that individuals with mismatched objective and subjective health statuses might be associated with worse health outcomes than those with matched health statuses.^20^ Past research has categorized individuals into three groups, including health realists (i.e., people’s perceptions match their actual health), health optimists (i.e., people’s perceptions are better than their actual health), and health pessimists (i.e., people’ perceptions are worse than their actual health). Given the potential power of unrealistic optimism about health,^21^ past research has proposed and demonstrated that health realists and optimists have lower mortality rates compared to their counterparts who are health pessimists.^20^ Similar patterns have also been observed for mental health outcomes.^22^ Some scholars have stressed that health congruence might also apply to weight congruence.^10^

To our best knowledge, only one study has formally assessed the effect of (mis)matched weight and weight perceptions on adolescents’ mental health. Using the National Longitudinal Study of Adolescent Health (Add Health) data, Frisco and colleagues found that normal weight adolescent boys with either overweight or underweight perceptions were more likely to report depressive symptoms compared to their counterparts who were normal weight with normal weight perceptions.^10^ Similar patterns had also been found among normal weight adolescent girls with either overweight or underweight perceptions.^10^ Together, these findings are consistent with the prediction of weight congruency perspective.^10^ Our study builds on Frisco and colleagues’ research^10^ by exploring three additional health and well-being outcomes including self-rated health, mental health, and life satisfaction.

## Methods

### Design

We used the 2017-2018 Canadian Community Health Survey (CCHS)—a nationally representative cross-sectional survey collected by Statistics Canada—to examine the research question. CCHS aims to characterize the health status, determinants of health, along with health care utilization patterns among Canadians aged 12 and older.^23^ The survey excluded residents of Indian reserves, health care institutions, some remote areas, and full-time members of the Canadian Forces.^23^ Both telephone and personal interviews were used to collect data, and each interview had an entry component, a health content, as well as an exit component.^23^ See Statistics Canada for further details about CCHS sample design and data collection procedures.^23^ The original sample of public use microdata file was 113,290 and the response rate was 60.8%.

### Sample

The present study only selected adolescents aged between 12 and 17. 8,654 adolescents were included, with a response rate of 53.4%.^23^ Due to small cell sizes, we excluded the following adolescents: 28 adolescents reported having post-secondary certificate diplomas and 90 adolescents reported having underweight and underweight perceptions, underweight and overweight perceptions, or overweight and underweight perceptions. Weight and weight perceptions had the most missing values (18.40% and 13.32%), followed by life satisfaction (5.03%) and mental health (4.89%). All other remaining variables had less than 2% of missing values. We applied multiple imputation techniques to deal with the missing values, where we included the dependent variables in the imputation procedure, but the analyses excluded cases with missing values on the dependent variables.^24^ Given that weight and weight perceptions had missing values, between 10% and 20%, we performed 20 imputations.^25^ The final sample included 8,081 adolescents.

### Measures

#### Self-rated health

was measured based on the following question: “In general, would you say your health is…?” The responses were recoded as “poor” (1), “fair” (2), “good” (3), “very good” (4), and “excellent” (5). Self-rated health has been found to be highly correlated with more objective measures of health status such as morbidity^26^ and mortality.^27^ It is also evident that self-rated health predicts outcomes more accurately compared to physician diagnoses.^28,29^ By following recent studies, we treated self-rated health as a continuous variable.^30-32^

#### Self-rated mental health

was measured based on the following question: “In general, would you say your mental health is…?” The responses were recoded as “poor” (1), “fair” (2), “good” (3), “very good” (4), and “excellent” (5). Self-rated mental health has been considered a useful measure of overall mental health. Although it is not a substitute measure of specific disorders, self-rated mental health and a variety of mental disorders (e.g., depression, psychological distress) are highly correlated; more importantly, self-rated mental health can capture both mental illness and mental well-being in a single-item question.^33,34^ By following recent studies, we treated self-rated mental health as a continuous variable.^33,35-37^

#### Self-rated life satisfaction

was measured based on the following question: “How do you feel about your life as a whole right now?” Responses were coded from “0” (very dissatisfied) to “10” (very satisfied). This single-item measure has shown to be a reliable and valid assessment of general well-being and has been widely employed in prior research.^17,38^

#### (Mis)matched weight and weight perceptions

We used two items to measure (mis) matched weight and weight perceptions. First, *weight* was measured based on weight (in kg) and height (in meters). The weight variable in the 2017-2018 CCHS was assessed based on WHO classification,^23^ including “thinness” (1), “normal” (2), “overweight” (3), and “obese” (4). To match the categories of weight perceptions, we combined categories of overweight and obese together. Second, *weight perceptions* were measured based on the following question “Do you consider yourself…?” Responses included “overweight” (1), “underweight” (2), and “just about right” (3). (Mis)matched weight and weight perceptions included 6 categories, which were presented above in Table 1. More specifically, matched categories were “normal weight and normal weight perceptions” (reference) and “overweight and overweight perceptions” while mismatched categories were “normal weight and underweight perceptions,” “underweight and normal weight perceptions,” “overweight and normal weight perceptions,” and “normal weight and overweight perceptions.”

#### Control variables

Based on past research, we controlled for the following socio-demographic variables because they could confound the association between weight/weight perceptions and health and well-being.^10,17^ *Age group* was measured as “aged between 12 and 14” (1) and “aged between 15 and 17” (2). *Family structure* was recoded as “child living with a single parent with or without siblings” (1), “child living with two parents with or without siblings” (2), and “other” (3). *Education* was recoded as “less than secondary school graduation” (1) and “secondary school graduation, no post-secondary education” (2). *Cultural/racial background* was measured as “White” (1),” “non-White” (2), and “Aboriginals” (3). *Total household income* was measured as “no income or less than $20,000” (1), “$20,000 to $39,999” (2), “$40,000 to $59,999” (3), “$60,000 to $79,999” (4), “$80,000 or more” (5). *Region* was recoded as “Atlantic” (1), “Central” (2), “Prairie” (3), “West” (4), and “Northern territories” (5). Table 2 presents the descriptive statistics of selected variables in the analysis.

**Table 2.** Descriptive Statistics for the Selected Variables in the Analysis.

	Boys (n = 4,088)		Girls (n = 3,993)	
	Mean/%	SE	Mean/%	SE
Self-rated health	4.06	.02	4.06	.02
Self-rated mental health	4.17	.02	3.95	.03
Self-rated life satisfaction	8.72	.03	8.66	.04
(Mis)matched weight and weight perceptions				
Underweight and normal weight perceptions	1.85		1.47	
Normal weight and overweight perceptions	1.29		2.86	
Normal weight and underweight perceptions	8.12		3.29	
Normal weight and normal weight perceptions (REF)	58.72		71.10	
Overweight and overweight perceptions	9.20		7.33	
Overweight and normal weight perceptions	21.09		14.41	
Age group				
12-14 (REF)	51.69		51.13	
15-17	48.31		48.87	
Family structure				
Child living with a single parent with or without siblings	18.90		20.37	
Child living with two parents with or without siblings (REF)	70.49		70.07	
Other	10.61		9.56	
Education				
Less than secondary school graduation (REF)	96.30		96.14	
Secondary school graduation, no post-secondary education	3.70		3.86	
Cultural/racial background				
White (REF)	64.02		65.07	
Non-white	30.75		29.23	
Aboriginals	5.23		5.70	
Total household income				
No income or less than $20,000	5.31		5.84	
$20,000 to $39,999	10.63		9.76	
$40,000 to $59,999	11.37		11.78	
$60,000 to $79,999	12.25		10.69	
$80,000 or more (REF)	60.44		61.93	
Region				
Atlantic	5.83		6.05	
Central (REF)	61.34		61.10	
Prairie	19.82		19.78	
West	12.61		12.67	
Northern territories	.40		.40	

Note: Means and percentages are weighted. “REF” refers to “the reference category” being used in the analysis.

### Analysis

The analysis included the following two steps. First, Table 3 tested the effects of weight and weight perceptions on self-rated health, mental health, and life satisfaction for adolescent boys and girls, respectively (OLS regression models). Second, Table 4 tested the effect of (mis)matched weight and weight perceptions on self-rated health, mental health, and life satisfaction for adolescent boys and girls, respectively (OLS regression models).

## Results

Table 3 presents OLS regression models of self-rated health, mental health, and life satisfaction on weight and weight perceptions. Models 1a and 1b—Models 3a and 3b focused on the effect of weight and Models 1c and 1d—Models 3c and 3d focused on the effect of weight perceptions. Model 1a showed that the levels of self-rated health did not differ between underweight and normal weight adolescent boys (b = .099, p > .05). However, overweight adolescent boys were associated with poorer self-rated health compared to their normal weight counterparts (b = −.202, p < .01). Model 1b showed similar patterns among adolescent girls, suggesting that only overweight adolescent girls were associated with poorer self-rated health compared to their normal weight counterparts (b = −.145, p < .01) and that the levels of self-rated health did not differ between underweight and normal weight adolescent girls (b = .154, p > .05). Models 2a and 2b showed that weight and self-rated mental health were unrelated among adolescent boys and girls. Likewise, weight and self-rated life satisfaction were unrelated among adolescent boys and girls as shown in Models 3a and 3b.

**Table 3.** OLS Regression Models of Self-Rated Health, Mental Health, and Life Satisfaction on Weight and Weight Perceptions.

	Self-rated health				Self-rated mental health				Self-rated life satisfaction			
	Boys	Girls	Boys	Girls	Boys	Girls	Boys	Girls	Boys	Girls	Boys	Girls
	M1a	M1b	M1c	M1d	M2a	M2b	M2c	M2d	M3a	M3b	M3c	M3d
	b	b	b	b	b	b	b	b	b	b	b	b
Weight												
Normal weight (REF)												
Underweight	.099	.154			.026	.086			.002	.104		
	(.155)	(.153)			(.174)	(.165)			(.300)	(.232)		
Overweight	−.202***	−.145**			−.044	−.080			−.095	−.113		
	(.050)	(.051)			(.048)	(.062)			(.068)	(.081)		
Weight perceptions												
About right (REF)												
Underweight			−.109	−.255*			−.250**	−.337*			−.222	−.323
			(.096)	(.118)			(.091)	(.170)			(.141)	(.200)
Overweight			−.540***	−.464***			−.303***	−.459***			−.591***	−.662***
			(.079)	(.068)			(.074)	(.077)			(.101)	(.122)
Intercept	4.154	4.203	4.147	4.211	4.316	4.233	4.344	4.254	8.893	8.925	8.922	8.951
N	4,088	3,993	4,088	3,993	4,088	3,993	4,088	3,993	4,088	3,993	4,088	3,993

Note: All models included a full set of control variables, including age group, family structure, education, cultural/racial background, total household income, and region. All coefficients were weighted. Standard errors were in parentheses. ***p < .001, **p < .01, *p < .05.

Model 1c in Table 3 also showed that adolescent boys with overweight perceptions were associated with poorer self-rated health compared to their counterparts with normal weight perceptions (b = −.540, p < .001). Model 1d showed a similar pattern among adolescent girls with overweight perceptions (b = −.464, p < .001), though adolescent girls with underweight perceptions were also associated with poorer self-rated health (b = −.255, p < .05). Model 2c showed that adolescents boys with either overweight (b = −.303, p < .001) or underweight (b = −.250, p < .01) perceptions were likely to reported poorer self-rated mental health compared to their counterparts with normal weight perceptions. Likewise, adolescent girls with either overweight (b = −.459, p < .001) or underweight (b = −.337, p < .05) perceptions were also associated with poorer self-rated mental health, as shown in Model 2d. Similar patterns had also been observed for self-rated life satisfaction, suggesting that adolescents with overweight perceptions were associated with poorer self-rated life satisfaction compared to their counterparts with normal weight perceptions (b = −.591, p < .001 for boys; b = −.662, p < .001 for girls), as shown in Models 3c and 3d.

Table 4 presents OLS regression models of self-rated health, mental health, and life satisfaction on (mis)matched weight and weight perceptions. Model 1a showed that normal weight adolescent boys with overweight perceptions were associated with poorer self-rated health compared to their normal weight counterparts with normal weight perceptions (b = −.541, p < .01). Similarly, overweight adolescent boys with overweight perceptions were associated with poorer self-rated health compared to normal weight counterparts with normal weight perceptions (b = −.546, p < .001). Model 1b showed similar patterns among adolescent girls, suggesting that normal weight adolescent girls with overweight perceptions (b = −.447, p < .001) and overweight adolescent girls with overweight perceptions (b = −.476, p < .001) were associated with poorer self-rated health compared to their normal weight counterparts with normal weight perceptions.

**Table 4.** OLS Regression Models of Self-Rated Health, Mental Health, and Life Satisfaction on (Mis)matched Weight and Weight Perceptions.

	Self-rated health		Self-rated mental health		Self-rated life satisfaction	
	Boys	Girls	Boys	Girls	Boys	Girls
	M1a	M1b	M2a	M2b	M3a	M3b
	b	b	b	b	b	b
(Mis)matched weight and weight perceptions						
Normal weight and normal weight perceptions (REF)						
Underweight and normal weight perceptions	.114	.163	.025	.102	.027	.076
	(.159)	(.148)	(.174)	(.165)	(.304)	(.221)
Normal weight and overweight perceptions	−.541**	−.447***	−.357	−.447**	−.727+	−.831***
	(.205)	(.133)	(.241)	(.150)	(.403)	(.223)
Normal weight and underweight perceptions	−.138	−.286*	−.232*	−.328+	−.129	−.366+
	(.100)	(.116)	(.096)	(.187)	(.141)	(.205)
Overweight and overweight perceptions	−.546***	−.476***	−.278***	−.433***	−.544***	−.617***
	(.088)	(.079)	(.080)	(.089)	(.105)	(.134)
Overweight and normal weight perceptions	−.070	.004	.027	.070	.066	.079
	(.054)	(.061)	(.057)	(.076)	(.079)	(.108)
Intercept	4.162	4.208	4.336	4.239	8.902	8.939
N	4,088	3,993	4,088	3,993	4,088	3,993

Note: All models included a full set of control variables, including age group, family structure, education, cultural/racial background, total household income, and region. All coefficients were weighted. Standard errors were in parentheses. ***p < .001, **p < .01, *p < .05, +p < .10.

Model 2a in Table 4 showed that overweight adolescent boys with overweight perceptions were associated with poorer self-rated mental health compared to their normal weight counterparts with normal weight perceptions (b = −.278, p < .001). A similar pattern had also been observed among adolescent girls (b = −.433, p < .001) as shown in Model 2b, though normal weight adolescent girls with overweight perceptions were also associated with poorer self-rated mental health (b = −.447, p < .01). Models 3a and 3b also showed similar patterns for self-rated life satisfaction, suggesting that overweight adolescents with overweight perceptions were associated with poorer self-rated life satisfaction (b = −.544, p < .001 for boys; b = −.617, p < .001 for girls) and that normal weight adolescent girls with overweight perceptions were associated with poorer self-rated life satisfaction (b = −.831, p < .001).

## Discussion

Although a large body of research has linked weight and weight perceptions to adolescents’ health and well-being, little is known about the extent to which (mis)matched weight and weight perceptions predict health and well-being disparities among adolescents. Using a nationally representative sample, we selected Canadian adolescents aged between 12 and 17 to examine how weight and weight perceptions jointly shape self-rated health, mental health, and life satisfaction.

Several findings reported in the present study are noteworthy. First, we found that overweight adolescents were associated with poorer self-rated health compared to their normal weight counterparts. This finding is consistent with prior research, suggesting that overweight adolescents are more likely to engage in unhealthy lifestyle behaviors, thereby leading to worse physical health outcomes.^12^ However, we found little evidence that weight and self-rated mental health and life satisfaction were related. These patterns suggest that actual weight might play a less important role in determining adolescents’ mental health and well-being.^9^ Second, we found that adolescents with overweight perceptions were associated with poorer self-rated health, mental health, and life satisfaction compared to their counterparts with normal weight perceptions. These findings are consistent with the prediction that the negative self-evaluation of being overweight influenced by social norms and cultural values is detrimental to adolescents’ health and well-being.^10,15^ Our findings align with the evidence presented in other studies,^7-9^ suggesting that weight perceptions are indeed more crucial than actual weight in determining adolescents’ health and well-being. Third, most research to date has focused on the effect of overweight perceptions on adolescents’ health and well-being consequences,^7,8^ our study discovered that adolescents with underweight perceptions were also associated with poorer self-rated mental health compared to their counterparts with normal weight perceptions. Past research has found a similar pattern. For instance, Frisco and colleagues^10^ found that adolescent boys and girls with underweight perceptions were more likely to report depressive symptoms. One possible explanation for this observation is that underweight perceptions could be conceptualized as a stigmatized status for adolescent boys because of their expectations on building and maintaining larger masculine body shape.^39^ However, it is unclear regarding why adolescent girls with underweight perceptions would be associated with poorer self-rated mental health. Future research should explore the potential mediating mechanisms embedded in that relationship.

Perhaps the most novel findings in the present study involve the ways in which weight and weight perceptions jointly shape adolescents’ self-rated health, mental health, and life satisfaction. On the one hand, we found that overweight adolescents with overweight perceptions were associated with poorer self-rated health, mental health, and life satisfaction compared to their counterparts with normal weight and normal weight perceptions. These patterns are consistent with the prediction of double jeopardy perspective, suggesting that weight and weight perceptions each represent a stigmatized social status, which contributes to an accumulation of stressors, thereby leading to greater health and well-being disparities.^18,19^ On the other hand, we observed that normal weight adolescents with overweight perceptions were associated with poorer self-rated health, mental health, and life satisfaction, except for self-rated mental health among adolescent boys. Overall, these patterns are consistent with the prediction of weight congruency,^10^ suggesting that mismatched objective weight and subjective weight perceptions are associated with greater health and well-being problems. Together, the present study found evidence supporting both double jeopardy and weight congruency perspectives. This is an important contribution for the following reasons. First, consistent with past research,^10^ our findings demonstrate that treating weight and weight perceptions as independent predictors might lead to an incomplete picture regarding the associations among weight, weight perceptions, and health and well-being consequences among adolescents. For instance, normal weight adolescent girls were not immune to adverse self-rated health, mental health, and life satisfaction because their weight perceptions also contributed to health and well-being outcomes. Second, with one exception,^10^ double jeopardy^18,19^ and weight congruency^20^ perspectives have not been formally applied to the health consequences of weight and weight perceptions. Our findings suggest that these theoretical aspects are indeed useful in predicting adolescents’ health and well-being by weight and weight perceptions.

Although the present study makes important contributions to the literature on health and well-being consequences among adolescents, there are also limitations. The first involves the potential reverse causality. We recognize the possibility that adolescents with poorer health and well-being might be more likely to develop negative self-evaluation toward their weight. However, given the ways that our hypotheses were grounded in theoretical perspectives, we do not see a particularly compelling case suggesting a reverse causality. Second, due to small cell sizes, we were unable to assess whether the effects of weight and weight perceptions on each health and well-being outcome further differs across social-demographic statuses such as cultural/racial background or household income. Third and finally, weight status employed in the present study was self-reported, which might be subject to reporting bias. Although some scholars posit that adolescent girls might be more likely to underestimate their weight compared to adolescent boys,^39^ there is evidence that self-reported weight status is reliable and valid,^40^ especially when the statistical models control for basic socio-demographic characteristics.^41^

Despite these limitations, the present study is among the first that formally assesses the extent to which weight and weight perceptions jointly shape multiple health and well-being consequences among adolescents. In addition to replicating our cross-sectional findings, we propose that future studies should examine the relationship from a life course perspective. Given that greater maturity, experience, and composure might weaken the detrimental effect of (mis)matched weight and weight perceptions, it is, therefore, important to empirically document health and well-being disparities by (mis)matched weight and weight perceptions over time.

As we have established the adverse association between (mis)matched weight and weight perceptions and health and well-being, it is critical for public health policy makers who aim at improving adolescents’ health and well-being to take actions. We encourage them to allocate more resources to explore the potential mediating or moderating mechanisms embedded in the association. For instance, normal weight adolescent girls with overweight perceptions might experience low self-esteem, which contributes to greater health and well-being disparities. Thus, improving self-esteem might reduce the harmful effect of (mis)matched weight and weight perceptions. Alternatively, their adverse health and well-being consequences might be lessened if normal weight adolescent girls with overweight perceptions have greater access to social support. Thus, providing parental and peer support might buffer against the detrimental effect of (mis)matched weight and weight perceptions.

**So What?**

**What is already known about this topic?**

Prior research has well-documented the effect of overweight/obesity on adolescents’ health and well-being. Some scholars have also recognized the importance of weight perceptions while assessing adolescents’ health and well-being disparities.

**What does this article add?**

Scholars have yet to explore whether (mis)matched weight and weight perceptions might be linked to greater health and well-being disparities among adolescents.

**What are the implications for health promotion practice or research?**

Our findings demonstrate that treating weight and weight perceptions as independent predictors might lead to an incomplete picture regarding the associations among weight, weight perceptions, and health and well-being among adolescents. For instance, normal weight adolescent girls are not immune to adverse self-rated health, mental health, and life satisfaction consequences because their weight perceptions also contribute to health and well-being. We encourage public health practitioners to explore the potential mediating or moderating mechanisms embedded in this adverse association. For instance, normal weight girls with overweight perceptions might experience low self-esteem, which contributes to greater health and well-being disparities. Thus, improving self-esteem might reduce the harmful effect of (mis)matched weight and weight perceptions. Alternatively, their adverse health and well-being consequences might be lessened if normal weight adolescent girls with overweight perceptions have greater access to social support. Thus, providing parental and peer support might buffer against the detrimental effect of (mis)matched weight and weight perceptions.
